# Comparative evaluation of the antioxidant, antimicrobial and nutritive properties of gluten-free flours

**DOI:** 10.1038/s41598-021-89845-6

**Published:** 2021-05-17

**Authors:** Joanna Miedzianka, Katarzyna Drzymała, Agnieszka Nemś, Agnieszka Kita

**Affiliations:** 1 Department of Food Storage and Technology, Faculty of Biotechnology and Food Science, Wroclaw University of Environmental and Life Sciences, Chełmońskiego 37, 51-630 Wrocław, Poland; 2 Department of Biotechnology and Food Microbiology, Faculty of Biotechnology and Food Science, Wroclaw University of Environmental and Life Sciences, Chełmońskiego 37, 51-630 Wrocław, Poland

**Keywords:** Biological techniques, Plant sciences

## Abstract

Gluten-free flours are interesting alternative to wheat flours. They could be by-products of oilseed processing, characterized by high content of bioactive compounds. Therefore the aim of the study was to determine the antioxidant, antimicrobial properties, amino acid and fatty acid profile of flours obtained as by-products from the oil industry. The highest total polyphenol content and antioxidant activity was found to have evening primrose flour. The widest spectrum of microbial growth inhibition was indicated for corn germ extract which showed no antimicrobial activity only against *Bacillus subtilis*. The highest protein content was found in pumpkin, peanut and almond flours (more than 50 g/100 g). The major abundant amino acids in all the analysed oilseed cake flours were aspartic acid, glutamic acid and arginine. The analysed gluten-free flours were found to be a good source of polyunsaturated fatty acids (PUFAs), which comprised mainly linoleic acid and α-linolenic acid, whereas the best source of PUFAs was evening primrose flour. The results suggest that the cold-pressed seed flours possess valuable chemical composition and may be considered for improvement of the nutritional properties of food products.

## Introduction

The most popular flour is wheat one, due to widely used in bakery products mainly thanks to formation of a viscoelastic dough. However, the gluten protein fraction responsible for it cannot be tolerated by patients suffering from coeliac disease. Yet, there are a number of flours available, in which gluten do not occur, and therefore they are safe to use even in these patients^1^.

One unconventional example of gluten-free flours are these obtained from oilseed cakes. They refer to the by-products after the oil extraction from the oilseed. Not so long ago, they have been used mainly as a feed component. However, thanks to the high nutritional value of these flours, closely related to the chemical composition of the raw material from which they come, as well as growing conditions and extraction methods^2^, they can also be a valuable component of food products. It is worth to emphasize that flours from oilseed cakes are rich source of good quality protein, deficient in lysine and sulphur amino acids, easily overcome by supplementation with other proteins and appropriate physico-chemical treatments, this protein could make a significant contribution to human dietary protein intake^3^. Moreover flours from oilseed cakes are characterised by a high content of fibre, minerals and vitamins, mainly from group B, and also possess a lower fat content than the raw materials^2,4^. They are rich in unsaturated fatty acids which are susceptible to hydrolytic and oxidative rancidity. The final product after cold pressing may also contain many valuable health-promoting ingredients, including phenolic compounds, tocopherols, squalene, sterols and carotenoids^5^. Therefore, the consumption of gluten free flours obtained as by-products from cold-pressed oils may improve human health and may prevent certain diseases.

Taking into account the organoleptic properties of gluten-free flours from oilseed cakes, they are characterised by interesting qualities which, along with good physico-chemical properties, allows for their versatile culinary use. They can be used to enrich different food products or as a source for the production of different food ingredients by partial replacement of wheat flour in confectionery and bakery products. Pasta, muffins and tortillas were prepared from a mixture of peanut flour and wheat flour. Pumpkin seed flour is used as an addition to wheat flour when baking bread, as well as for baking cakes, breading or thickening soups. Bread with the addition of flax flour maintains its humidity longer than wheat bread, which significantly extends its freshness. Peanut flour is not only a great addition to bread as a gluten-free substitute, but it is also used to prepare noodles, pasta and various sweet pastries^6^.

In the literature there is available much information on the chemical composition of flours from oilseed cakes but data on the antimicrobial properties is scarce. Moreover, it is worth to analyse the antioxidant properties of flours from oilseed cakes as during the oil extraction process, most of the tocopherols and some of the phospholipids are removed^7^. Several reports exist on the nutritive values of proteins and oils from oilseed cakes^3,8^. Some concentrated on the removal of the anti-nutritional factors present in the oilseed cakes^9^. Due to the fact that processes such as dehulling and milling significantly change the nutrient profile, the characterisation of such flours is interesting. The aim of this study was to characterise the nutritive properties with amino acid and fatty acid profile as well as antimicrobial and antioxidant properties of unconventional gluten free flours made from oilseed cakes from pumpkin, flax, evening primrose, milk thistle, corn germ, almond and peanut. These results could provide information about potential application of the studied flours to substitute or complement wheat flour in new food products where these by-products could be used in the food industry to achieve economic gain^10^.

## Results

### Phenolic content and antioxidant activity

**Figure 1:**
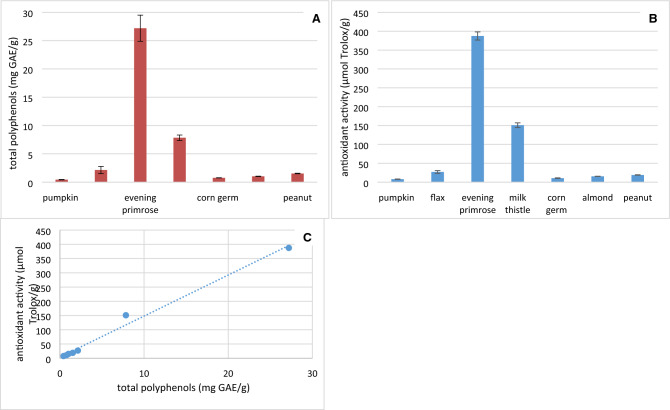
Total polyphenols (**A**) content, antioxidant activity (ABTS˙^+^) (**B**) and correlation between total polyphenols content and antioxidant activity (**C**) of selected cold-pressed seed flours.

Phenolic compounds are secondary metabolites naturally present in vegetable material and have been associated with their antioxidant and antimicrobial properties. The total phenolic content of the cold-pressed seed flours analysed here ranged from 0.43 to 27.20 mg gallic acid equivalent (GAE)/g, whereas the antioxidant activity, determined on the basis of the 2,2′-azino-bis-3-ethylbenzothiazoline-6-sulphonic acid (ABTS˙^+^), ranged from 7.77 to 387.39 µmol Trolox equivalent antioxidant capacity (TEAC)/g (Fig. 1A,B, respectively). Evening primrose seed flour was found to have the highest total polyphenols content and the antioxidant activity. Subsequently, the highest content of total polyphenols and antioxidant activity was found in milk thistle flour, followed by flax, peanut, almond, corn and pumpkin seeds in descending order. Moreover, based on the present studies, there is a strong positive correlation (0.99) between the antioxidant activity and total polyphenol content of the flours analysed (Fig. 1C).

### Antimicrobial activity

The potential antimicrobial activity of methanol extract of gluten-free flours obtained from by-products from the oil industry was evaluated by the diffusion method. Results summarized in Table 1 indicate that all oilseed samples exhibited an antimicrobial activity at different extents. The widest spectrum of microbial growth inhibition was indicated for corn germ extract, which showed no antimicrobial activity only against *Bacillus subtilis.* Corn germ presented similar and strong inhibition of growth for the remaining Gram-positive (Gram+) and Gram-negative (Gram−) bacteria. In this study, corn germ extract was additionally tested against the yeast *Candida albicans*; it is noteworthy that the result achieved (6.58 mm) was the largest area of growth inhibition demonstrated in this research. Activity against Gram+ bacteria was also shown for evening primrose extract—stronger (6.35 mm) against *Enterococcus faecalis* and weaker (4.45 mm) against *Enterococcus hirae*. Effective growth inhibition of *E. faecalis*, although over a significantly smaller area (1.89 mm), was observed here for milk thistle extract. Lastly, peanut extract was, like corn germ extract, effective against *Vibrio harveyi*, showing growth inhibition zones of 4.63 and 5.30 mm respectively. No microbial growth inhibition was indicated for pumpkin, flax and almond seed flours. Additionally, in analysed oilseed flours, a positive correlation was obtained between total polyphenol content and antimicrobial activity of corn germ, milk thistle and evening primrose against faecalis (r = 0.66).

**Table 1:** Zones of inhibition (mm) in the growth of microorganism of selected cold-pressed seed flours.

Flours	G-positive bacteria (G+)				G-negative bacteria (G−)			Yeast
	*Staphylococcus aureus*	*Bacillus subtilis*	*Enterococcus hirae*	*Enterococcus faecalis*	*Escherichia coli*	*Pseudomonas aeruginosa*	*Vibrio harveyi*	*Candida albicans*
Pumpkin	–	–	–	–	–	–	–	–
Flax	–	–	–	–	–	–	–	–
Evening primrose	–	–	4.45 ± 0.84	6.35 ± 0.09	–	–	–	–
Milk thistle	–	–	–	1.89 ± 0.22	–	–	–	–
Corn germ	6.28 ± 0.99	–	2.67 ± 0.99	5.77 ± 0.36	6.15 ± 1.18	5.09 ± 0.50	5.30 ± 1.36	6.58 ± 0.50
Almond	–	–	–	–	–	–	–	–
Peanut	–	–	–	–	–	–	4.63 ± 0.40	–

– Not identified (trace or % < 0.1).

### Proximate composition

The samples of gluten-free flours from cold-pressed seeds analysed here were characterised by a similar, narrow range content of dry matter (DM), valued from 90.39 g/100 g to 96.64 g/100 g (Table 2). Statistically, the lowest water content was found in peanut oilseed flour (3.36 g/100 g), whereas the highest content was noted in flax flour (9.61 g/100 g). The total ash content of the flours analysed was significantly higher (from 3.43 g/100 g for flax to 8.44 g/100 g for pumpkin seed flour) as compared to wheat flour (about 1 g/100 g). The selected cold-pressed seed flours are characterised by a lack of gluten and a higher protein content (Table 2) as compared to wheat bread flours. The highest protein content was found in pumpkin, peanut and almond flours (more than 50 g/100 g), whereas the lowest was found in corn germ flour (16 g/100 g). The cold-pressed seed oil flours analysed here have a high fat content. The highest fat content was found in flax seed flour (14.24 g/100 g), whereas the lowest was found in peanut flour (6.78 g/100 g). Two different types of carbohydrate are present in oilseed flours: soluble mono- and oligosaccharides as well as insoluble polysaccharides, which play a significant role in determining the functional properties. Selected cold-pressed flours were characterised by a lower carbohydrate content compared to wheat flour (72 g/100 g). The corn germ oilseed flour was characterised by the highest carbohydrate content (62.71 g/100 g), and the lowest was found in pumpkin seed flour (11.12 g/100 g). The crude fibre content of the oil seed by-products varied between 3.21 and 36.28 g/100 g, as shown in Table 2. Among the by-products analysed, evening primrose and milk thistle flour were good sources of crude fibre, containing six- to nine-fold more fibre than corn germ, pumpkin and peanut samples.

**Table 2:** Chemical composition (g/100 g) of selected cold-pressed seed flours.

Content	Flour						
	Pumpkin seeds	Flax	Evening primrose	Milk thistle	Corn germ	Almond	Peanut
Dry matter	94.44 ± 0.02^c^	90.39 ± 0.01^f^	92.52 ± 0.05^f^	94.05 ± 0.01^d^	93.09 ± 0.01^e^	96.38 ± 0.06^b^	96.64 ± 0.11^a^
Ash	8.44 ± 0.02^a^	3.43 ± 0.01^c^	7.56 ± 0.02^a^	7.12 ± 0.03^ab^	5.36 ± 0.01^bc^	6.44 ± 0.02^ab^	3.62 ± 0.01^bc^
Protein	66.76 ± 2.31^a^	36.06 ± 0.74^d^	22.79 ± 0.47^f^	26.45 ± 0.62^e^	16.13 ± 0.91^g^	50.19 ± 1.17^c^	57.88 ± 2.47^b^
Fat	8.12 ± 0.04^d^	14.24 ± 0.16^a^	9.98 ± 0.18^b^	9.71 ± 0.24^b^	8.89 ± 0.31^c^	10.10 ± 0.21^b^	6.78 ± 0.14^e^
Carbohydrates	11.12 ± 1.03^e^	36.66 ± 0.65^c^	52.19 ± 0.28^b^	50.77 ± 0.51^b^	62.71 ± 0.37^a^	29.65 ± 0.65^d^	28.36 ± 1.00^d^
Crude fibre	4.39 ± 0.35^ef^	12.01 ± 0.04^c^	36.28 ± 1.75^a^	32.35 ± 1.02^b^	3.21 ± 0.42^f^	8.26 ± 0.11^d^	5.45 ± 0.17^e^

Values are means ± SD of two determinations. ^a,b,c,d,e,f^The same letter in verse mean homogenous groups.

### Amino acids

The amino acid composition of cold-pressed seed by-products is presented in Table 3. Total essential amino acids differed significantly, ranging from 3.47 g/100 g in corn germ flour to 14.51 g/100 g in pumpkin seed flour. The major abundant amino acids were aspartic acid, glutamic acid and arginine, their sum ranging from 3.01 (corn germ oil seed flour) to 21.78 g/100 g (pumpkin flour). Based on the amino acid profile of cold-pressed seed flours, all analysed samples contained incomplete protein. The amino acid most limiting the nutritional value in selected flours was lysine (Lys), except for peanut flour in which threonine (Thr) was found. Diversity was observed in terms of the second most limiting amino acid. The biological value of selected samples was decreased by the content of leucine (Leu) (in pumpkin seed and almond flour), sulphuric amino acids (in flax and corn germ flours), Thr (in evening primrose) and Lys (in peanut flour).

**Table 3:** Amino acid concentration (g/100 g) of flours from oilseed cakes.

Amino acid	Pumpkin	Flax	Evening primrose	Milk thistle	Corn germ	Almond	Peanut	FAO/WHO reference pattern (1991)
**IAA**								
Leucine	3.43 ± 0.01^a^	1.66 ± 0.04^c^	1.40 ± 0.03^d^	0.90 ± 0.04^e^	0.86 ± 0.13^e^	2.41 ± 0.03^b^	1.60 ± 0.03^c^	6.60
Isoleucine	1.82 ± 0.05^a^	1.13 ± 0.01^c^	0.75 ± 0.14^d^	0.59 ± 0.13^e^	0.41 ± 0.03^f^	1.33 ± 0.04^b^	0.77 ± 0.01^d^	2.80
Methionine	1.01 ± 0.05^a^	0.59 ± 0.06^b^	0.41 ± 0.17^c^	0.31 ± 0.24^d^	0.21 ± 0.12^e^	0.31 ± 0.01^de^	0.23 ± 0.01^e^	
Cysteine	0.38 ± 0.00^a^	0.22 ± 0.03^b^	0.23 ± 0.02^b^	0.15 ± 0.02^c^	0.06 ± 0.06^e^	0.21 ± 0.02^b^	0.39 ± 0.01^a^	
Methionine + cysteine								2.50
Phenylalanine	2.51 ± 0.04^a^	1.36 ± 0.05^c^	1.07 ± 0.06^d^	0.65 ± 0.17^e^	0.60 ± 0.14^e^	1.94 ± 0.08^b^	1.01 ± 0.03^d^	
Threonine	1.34 ± 0.01^a^	0.95 ± 0.00^c^	0.52 ± 0.05^d^	0.48 ± 0.08^d^	0.38 ± 0.05^e^	1.01 ± 0.19^b^	0.18 ± 0.02^f^	
Phenyloalanine + threonine								3.40
Lysine	1.96 ± 0.02^a^	1.11 ± 0.02^b^	0.52 ± 0.00^e^	0.67 ± 0.04^d^	0.48 ± 0.62^e^	0.95 ± 0.20^bc^	0.84 ± 0.02^c^	6.30
Tyrosine	1.55 ± 0.00^a^	0.81 ± 0.03^cd^	0.67 ± 0.06^de^	0.61 ± 0.12^e^	0.50 ± 0.10^f^	1.13 ± 0.16^b^	0.80 ± 0.03^c^	5.80
Valine	2.45 ± 0.01^a^	1.44 ± 0.06^c^	1.01 ± 0.04^d^	0.69 ± 0.02^e^	0.53 ± 0.38^f^	1.67 ± 0.14^b^	1.33 ± 0.03^c^	3.50
**DAA**								
Aspartic acid	4.25 ± 0.02^a^	2.54 ± 0.05^c^	1.69 ± 0.11^d^	1.33 ± 0.26^f^	0.85 ± 0.09^h^	3.62 ± 0.04^b^	1.48 ± 0.08^e^	
Glutamic acid	8.95 ± 0.02^a^	4.82 ± 0.16^d^	3.84 ± 0.14^e^	2.14 ± 0.10^f^	1.30 ± 0.41^g^	8.20 ± 0.24^b^	6.70 ± 0.19^c^	
Serine	2.05 ± 0.00^a^	1.19 ± 0.02^c^	0.92 ± 0.09^d^	0.72 ± 0.08^e^	0.51 ± 0.17^f^	1.34 ± 0.18^b^	0.19 ± 0.01^g^	
Glycine	2.64 ± 0.01^a^	1.65 ± 0.01^c^	1.36 ± 0.02^d^	0.75 ± 0.05^e^	0.46 ± 0.05^g^	2.09 ± 0.02^b^	1.33 ± 0.02^d^	
Alanine	2.32 ± 0.01^a^	1.30 ± 0.02^d^	0.86 ± 0.02^e^	0.55 ± 0.07^h^	0.67 ± 0.14^f^	1.55 ± 0.04^c^	1.97 ± 0.02^b^	
Histidine	1.32 ± 0.00^a^	0.63 ± 0.00^c^	0.53 ± 0.06^c^	0.35 ± 0.07^d^	0.30 ± 0.40^d^	0.88 ± 0.08^b^	0.61 ± 0.02^c^	
Arginine	8.58 ± 0.10^a^	3.12 ± 0.10^c^	2.78 ± 0.08^d^	1.44 ± 0.11^e^	0.86 ± 0.19^f^	4.27 ± 0.13^b^	3.10 ± 0.06^c^	
Proline	1.66 ± 0.22^a^	1.04 ± 0.02^d^	0.41 ± 0.33^g^	0.59 ± 0.20^ef^	0.70 ± 0.16^e^	1.52 ± 0.11^b^	1.18 ± 0.07^c^	
I Limiting amino acid	Lys	Lys	Lys	Lys	Lys	Lys	Thr	
II Limiting mino acid	Leu	Met + Cys	Thr	Thr	Met + Cys	Leu	Lys	

Values are means ± SD of three determinations. ^a,b,c,d,e,f,g^The same letters within the same row were not significantly different. *IAA* indispensable amino acids, *DAA* dispensable amino acids.

### Fatty acids

The flours from cold-pressed seeds analysed here were characterised by a small content of saturated fatty acids (SFAs), ranging from 8.0% (almond flour) to 19.7% (milk thistle flour) (Table 4). The major SFAs found in all oilseed flours analysed were palmitic acid (C16:0) and stearic acid (C18:0). However, peanut flour was an abundant source of palmitic acid (12.47%) and pumpkin seed flour was a source of stearic acid (4.90%), as compared with other samples.

**Table 4:** Fatty acid composition (% of total fatty acid profile) of flours from oilseed cakes.

Fatty acid	Pumpkin	Flax	Evening primrose	Milk thistle	Corn germ	Almond	Peanut
Lauric acid (C12:0)	0.05 ± 0.01^b^	0.01 ± 0.01^c^	0.11 ± 0.01^a^	0.01 ± 0.01^c^	0.02 ± 0.01^c^	0.01 ± 0.01^c^	0.03 ± 0.01^bc^
Myristic acid (C14:0)	0.10 ± 0.0^a^	0.02 ± 0.01^c^	0.12 ± 0.02^a^	0.10 ± 0.01^a^	0.08 ± 0.02^a^	0.05 ± 0.01^b^	0.08 ± 0.02^a^
Palmitic acid (C16:0)	11.96 ± 0.12^b^	4.80 ± 0.02^e^	6.03 ± 0.11^d^	8.64 ± 0.05^c^	11.4 ± 0.11^b^	6.18 ± 0.08^d^	12.47 ± 0.11^a^
Palmitoleic acid (C16:1)	0.17 ± 0.02^c^	0.09 ± 0.01^d^	0.14 ± 0.02^c^	0.09 ± 0.02^d^	0.13 ± 0.02^c^	0.62 ± 0.02^a^	0.46 ± 0.01^b^
Stearic acid (C18:0)	4.90 ± 0.03^a^	3.62 ± 0.03^c^	1.83 ± 0.03^e^	4.72 ± 0.07^b^	1.10 ± 0.01^f^	1.63 ± 0.02^e^	2.79 ± 0.03^d^
Oleic acid (C18:1)	37.93 ± 0.13^c^	18.92 ± 0.09^f^	15.76 ± 0.12^g^	24.21 ± 0.13^e^	25.87 ± 0.09^d^	64.98 ± 0.28^a^	63.54 ± 0.21^b^
Linoleic acid (C18:2)	44.07 ± 0.22^d^	19.1 ± 0.02^g^	67.56 ± 0.26^a^	55.71 ± 0.24^c^	59.97 ± 0.19^b^	26.14 ± 0.12^e^	20.50 ± 0.09^f^
Linolenic acid (C18:3)	0.33 ± 0.01^d^	53.4 ± 0.14^a^	8.34 ± 0.09^b^	0.29 ± 0.02^e^	1.03 ± 0.03^c^	0.26 ± 0.04^e^	nd
Arachidic acid (C20:0)	0.32 ± 0.01^b^	0.03 ± 0.01^d^	0.06 ± 0.02^d^	3.50 ± 0.02^a^	0.3 ± 0.01^d^	0.08 ± 0.02^c^	0.05 ± 0.01^d^
Behenic acid (C22:0)	0.17 ± 0.01^b^	0.02 ± 0.01^e^	0.09 ± 0.01^c^	2.73 ± 0.03^a^	0.1 ± 0.01^e^	0.05 ± 0.01^d^	0.08 ± 0.02^c^
∑SFA	17.5	8.5	8.2	19.7	13.0	8.0	15.5
∑MUFA	38.1	19.0	15.9	24.3	26.0	65.6	64.0
∑PUFA	44.4	72.5	75.9	56.0	61.0	26.4	20.5

*MUFA* monounsaturated fatty acids, *PUFA* polyunsaturated fatty acids, *SFA* saturated fatty acids. Values are means ± SD of three determinations.

^a,b,c,d,e,f,g^The same letters within the same row were not significantly different.

The flours analysed in this study were characterised by a differential content of monounsaturated fatty acids (MUFAs), in the range 15.9–65.6%. The lowest content of these fatty acids was found in evening primrose flour, whereas almond and peanut flour were characterised by the highest MUFA content. They comprised mainly oleic acid (C18:1), the abundant sources of this fatty acid being almond and peanut flours (64.98% and 63.54%, respectively).

The cold-pressed seed flours analysed were found to be a good source of polyunsaturated fatty acids (PUFAs), their quantity ranging from 20.5% (peanut flour) to 75.9% (evening primrose flour). They comprised linoleic acid (C18:2) and α-linolenic acid (C18:3). Evening primrose flour was found to be the best source of linoleic acid (67.56%) and flax flour was characterised by the highest quantity of α-linolenic acid (53.4%), compared to other samples. Linolenic acid was undetected only in peanut oilseed flour.

## Discussion

### Phenolic content and antioxidant activity

The highest total polyphenols content and antioxidant activity was found for evening primrose seed flour, which is mainly related to three major low molecular weight phenolic compounds, namely, (+)-catechin, (−)-epicatechin and gallic acid and several flavonoids which have been identified so far^11,12^. Investigations on evening primrose polyphenol extracts and their biological activity have been limited to a few in vitro studies^11^ and one in vivo study^13^. Additionally, the phenolics present in evening primrose seed flour, constituting a large amount of waste material from the oil processing industry, are planned to be introduced on the market as a diet supplement in some countries. Whereas, the high antioxidant activity of milk thistle seed flour is related to flavonolignans (with silybin being the major one, and much more abundant than other phenolics). Also studies presented by Serçe et al.^14^ showed that milk thistle seeds have good antioxidant capacity using the 2,2-diphenyl-1-picrylhydrazyl (DPPH) method and can prevent lipid peroxidation. Moreover, differences have been noticed between flours from the same plants due to the seed genotype, crop growing conditions and/or seed storage conditions^5,15^. In comparison, cereal products can also be a source of antioxidants due to the scale of intake. The antioxidant activity of extracts from cereal products shows a correlation with the content of phenols in these products (Fig. 1C).

### Antimicrobial activity

Corn germ extract showed the widest spectrum of microbial growth inhibition, as compared to other analysed flours, against bacteria and yeast, except subtilis microorganisms. Similarly, Mahmoud et al.^16^ demonstrated growth inhibition for *Staphylococcus aureus, Salmonella enterica*, *Listeria monocytogenes* and *Escherichia coli* by wheat germ. Also antibacterial activity against *S. aureus* has previously been indicated for almond- and pomegranate-based by-products^17^ and for rosemary extracts^18^. Growth inhibition against *C. albicans* by corn germ extract is important as this yeast in normal healthy circumstances is a harmless organism; however, it can be a pathogen for people with immunological weakness and can be responsible for oral disease^19^. Based on the presented studies, there can be concluded that strong growth inhibition against faecalis and weaker against hirae was found also for evening primrose and milk thistle extracts. Enterococci are hospital-associated pathogens capable of causing a variety of infections. This result was confirmed by Choe et al.^8^ who demonstrated that milk thistle can be useful to reduce the growth of *Enterobacteriaceae*. On the basis of the presented results, it is possible to consider that some plant by-products could be a source of antimicrobial substances and activity. Moreover, high positive correlation obtained between total polyphenols content and antimicrobial activity of corn germ, milk thistle and evening primrose against faecalis could be resulted from phenolic acids, which are the main compounds representing the phenolic fraction in maize, wherein ferulic acid is the most abundant, followed by ρ-coumaric, and isoferulic, ρ-hydroxybenzoic, gallic, syringic and chlorogenic acids, quercetin, quercitrin, and kaempferol identified as the main flavonoids^20^.

### Proximate composition

One of the most important chemical content quality indicator is the amount of DM. In flours, a moisture content higher than 14% affects the storage quality as mould growth, insect infestation and agglomeration start to occur^21^. The samples of gluten-free flours from cold-pressed seeds were characterised by a low content of DM (Table 2), what is a good indication of microbial stability and may also contribute to reducing the tendency of baked food products to become stale^21^. Based on the high total ash content, as compared to wheat flours, the flours from cold-pressed seeds have the potential to increase the mineral intake in the diet when combined in food products with a low ash content. However, on the other hand, a high ash content is considered to represent poorer product quality due to the dilution effect on the functional proteins^3^.

Total protein content is an important indicator of the physico-chemical properties of flours; moreover, it affects the baking value. Wheat bread flours should have a protein content in the range 11–14%^21^. The selected cold-pressed seed flours are characterised by a lack of gluten and a higher protein content (Table 2). Almost all analysed flours, except the corn germ flour, may be considered as high-protein foods where at least 20% of the energy is provided by protein^9^. However, comparing the data with other studies found in the literature, the protein content found in pumpkin oilseed flour was different, which could have resulted in having a higher fat content. The addition of oilseed flours rich in protein to bread can increase the overall energy intake from protein as well as affecting the bread volume, due to the ability to bind water in the crumb, and this furthermore can lead to a decrease in the energetic volume of ready-to-eat products. Also, according to Lee et al.^22^, the introduction of pumpkin flour in the processing of noodles, breads and cakes can not only enhance the content of various nutrients but also improve the flavour of ready products. In conclusion, selected cold-pressed seed flours from pumpkin, almond and peanut might be used to produce enriched functional foods.

The high fat content in cold-pressed flours is related to the fat content of the raw material from which they originate (Table 2). On the other hand, the presence of a high fat content in flours can create a barrier to incorporating them into foods due to the lipoxygenase-catalysed oxidation of unsaturated fatty acids to volatile compounds, which leads to a deterioration of flavour and a reduction in the shelf-life of food products^9^. Flax seed flour was characterized by the highest fat content, which is also confirmed by Davis et al^2^. Furthermore, the studies presented by Cozea et al.^23^ revealed that there is a close correlation between proteins and lipids, due to the ability to facilitate the transfer of proteins from phospholipid cell membranes. It is therefore important to have an equilibrate ratio between these two compounds.

Cereal flours contain two types of polysaccharides: starch and NSP, which together with lignins are called dietary fibre. Among all analysed samples, the evening primrose flour was found to be the best source of dietary fibre (Table 2). Taking into account the recommendation from the European Food Safety Authority (EFSA) for consumption of more than 25 g of fibre a day, the cold-pressed by-products could be used as a new source for production of different dietary fibre types (including also milk thistle flour). Additionally, consumption of sufficient dietary fibre can, inter alia, reduce the risk of cardiovascular disease, colon cancer and obesity. Furthermore, products rich in dietary fibre have attracted attention as food ingredients and have encouraged researchers to search out new fibre sources. Taking into account that in recent years cellulose is considered as one of the major ingredients for developing products with a functional purpose, oil cakes with an increased cellulose content could be added to new formulations as a source of insoluble food fibre.

### Amino acids

The biological value of protein found in food, i.e., its usefulness for anabolic purposes, depends on the content of particular essential amino acids as well as on the sum of non-essential amino acids. Proteins from oilseed cakes are considered to be of poor quality compared to animal ones, which are perceived as the source of essential amino acids. Analysed oilseed flours were characterized by differential total essential amino acids (Table 3). This observation was in close agreement with that of Olaofe^24^ who analysed the amino acid composition of melon, pumpkin and gourd seeds. However, Aremu et al.^25^ when studying six Nigerian legume flours, found a total essential amino acid content higher than that of the samples analysed in this study, ranging between 39.3 and 48.3%. The major abundant amino acids in all oilseed samples were aspartic acid, glutamic acid and arginine. High arginine content in food products is important because it has a beneficial effect on people suffering from coronary artery disease and high blood pressure. It is also important for people who actively play sports, because it accelerates the regeneration of muscle cells due to their better oxygenation. In addition, it increases synthesis of the growth hormone somatotropin, responsible for the regeneration of the human body. On the basis of these studies, it is concluded that flours from oilseed by-products will not contribute significantly to the supply of essential amino acids in the diet. However, it is also worth emphasising that the amino acid composition of proteins from oilseeds, except for peanut and corn germ proteins, is generally more beneficial than the composition of wheat proteins, characterised by a lower content of such amino acids as lysine, phenylalanine, arginine and asparagine acid^26^.

Based on the amino acid profile of cold-pressed flours, they cannot be used as the only protein source in the diet, as they contain incomplete protein (Table 3). In most analysed samples, the first limiting amino acid is Lys. However, when eating plant food products, the deficiency of a specific essential amino acid in one protein can be supplemented with an amino acid found in large amounts in another protein. Additionally, using two or three different sources of vegetable protein, the need for essential amino acids can be fully satisfied. For example, the nutritional value of proteins from oilseeds can be increased by combining them with proteins from leguminous plants, in which methionine and cysteine are the limiting amino acids, or by adding to them even small amounts of full-value animal protein. It is worth emphasising that plant proteins are considered to have lower nutritional quality, mainly because of poorly digestible fractions, and a high content of anti-nutritional compounds (enzyme inhibitors, tannins or phytates). The method used to extract protein has a great influence on its digestibility. Some processing steps, like cooking or microwave treatment, have been found to increase protein digestibility.

### Fatty acids

Fatty acid composition is particularly important for the nutritional value of food products. In total, 10 fatty acids were detected, identified and quantified in total lipid extracts (Table 4). The major SFAs found in all oilseed flours analysed were palmitic and stearic acids. SFAs are important to nutrition because of their ability to elevate blood lipid levels in humans. Nutritional recommendations around the world suggest that SFA intake should be kept low. The diversification in analysed samples was found among MUFAs. They comprised mainly oleic acid. Karaman et al.^17^ noticed that cold-pressed edible oil by-products from almond are rich in MUFAs, in the range of 60.18%, as compared with samples from walnut, pomegranate and grape. As seen, our results were in accordance with findings in the literature. Among PUFAs fatty acids, the most abundant was linoleic and α-linolenic acids, where the best source of these fatty acids was evening primrose flour. Similar findings were presented by Montserrat-de la Paz et al.^27^ who found that evening primrose oil is very high in linoleic (70–74%) and γ-linolenic (8–10%) acids, and also contains other fatty acids: palmitic acid, oleic acid, stearic acid and (in smaller amounts) myristic acid, oleopalmitic acid, vaccenic acid, eicosanoic acid and eicosenoic acid. Linoleic acid belongs to the group of essential fatty acids and plays an important role in the proper functioning of the skin (prevents the skin from peeling and losing water through the epidermis), reduces the inflammatory reaction and eases eye problems such as burning and dryness. In comparison, according to Goesaert et al.^28^, the fatty acid pattern of wheat flour lipids is dominated by linoleic acid, with lower amounts of palmitic and oleic acids. The consumption of vegetable oils is currently increasing due to their high content of PUFAs which possess a natural preventive role in cardiovascular disease and promote the reduction of total and HDL cholesterol. Therefore, enrichment of different food products with these oil by-products could be suggested regarding their fatty acid composition, gaining not only economic but also health benefits.

## Materials and methods

### Materials and reagents

The subjects of the study were flour samples from the following oilseed cakes: pumpkin, flax, evening primrose, milk thistle, corn germ, almond and peanut, obtained from the Ol’Vita company (Myslakow, Poland). Flours were obtained by milling the seed cakes obtained during the cold-pressing production of oil. The ready products were packed into polypropylene bags and closed with a clip, leaving a small space filled with air above the sample. The weight of one package was 250 g. Samples were stored in the laboratory in their original, commercial packages at 22 ± 1 °C in the dark.

All reagents used were of analytical grade. Methanol, Folin–Ciocalteu reagent, 2,2′-azino-bis-3-ethylbenzothiazoline-6-sulphonic acid (ABTS), boron trifluoride (BF_3_) and diethyl ether were purchased from Sigma (St. Louis, MO, USA). Ninhydrin, hydrindantin, methylcellosolve and sodium acetate buffer were purchased from INGOS (Prague, Czech Republic).

### Determination of total phenolic and antioxidant activity

Total polyphenol content was determined using the Folin–Ciocalteu colorimetric method, as described by Gao et al.^29^ with some modifications. Briefly, the flour samples were extracted in 70% aqueous methanol in a graduated tube. The mixtures were homogenised using a vortex for 5 s, agitated in an ultrasonic bath for 30 min and shaken for 60 min. After 20 h of storage at − 22 °C, the prepared solutions were again shaken for 90 min and centrifuged (at 10,000 rpm for 10 min at 4 °C). The remaining extracts were brought to a known volume with 70% methanol and stored at − 20 °C until further analysis. Polyphenol content, expressed as milligrams of gallic acid equivalent (GAE), was calculated per gram of dry matter (DM). The antioxidant activity of aqueous extracts was determined using the Trolox equivalent antioxidant capacity (TEAC) with the ABTS method according to Re et al.^30^ using a Rayleigh UV-2601 PC spectrophotometer (Beijing, China). TEAC results were expressed as micromoles of Trolox equivalents per gram of DM. Data are reported as the mean value ± SD for three measurements.

### Antimicrobial activity

#### Preparation

The antimicrobial activity of flours from oilseed cakes was determined for the following microorganism strains: (1) Gram-positive bacteria (G+) *Staphylococcus aureus* (ATCC 9538), *Bacillus subtilis* (ATCC 6633), *Enterococcus faecalis* (ATCC 29212) and *Enterococcus hirae* (ATCC 10542); (2) Gram-negative bacteria (G−) *Escherichia coli* (ATCC 10536), *Vibrio harveyi* (ATCC 12126) and *Pseudomonas aeruginosa* (ATCC 15442); and (3) the yeast *Candida albicans* (ATCC 10231). All microorganisms were obtained from the culture collection of the Department of Biotechnology and Food Microbiology (WUELS, Wrocław). Bacteria were grown in Luria broth (Sigma, Germany; containing 10 g/L tryptone, 5 g/L yeast extract and 5 g/L NaCl) at 37 °C; yeast was grown in YPD broth (Sigma, Germany; containing 20 g/L bacteriological peptone, 10 g/L yeast extract and 20 g/L glucose) at 30 °C. Agar was added to the medium at a concentration of 2% when necessary. To prepare an inoculation culture for the agar diffusion method, the cultures were grown in a 0.1 L flask containing 10 mL of proper medium, on a rotary shaker at 37 or 30 °C at 180 rpm for 48 h. Cells were washed in saline solution and adjusted to OD600 = 1.

#### Extraction procedure

For the extraction of potential antimicrobial compounds, 1 g of sample was mixed with 10 mL of methanol, stirred by vortex and shaken for 2 h at ambient temperature. The solution was centrifuged at 5500×*g* for 5 min and the supernatants were sterilised by the filtration method with the use of a 0.22 µm syringe filter.

#### Determination of antimicrobial properties by agar diffusion assay

An agar diffusion assay (well diffusion assay) was used to test the antimicrobial activity of extracts. On each plate with proper medium, three wells were made—two containing 100 µL of the extract to be tested, and a third containing 100 µL of methanol as a control sample. All tests were carried out in triplicate. Agar plates were inoculated with a 200 µL of standardized to OD600 = 1 inoculum of the test microorganism and left at optimal temperature for 2 h. Subsequently, wells were made in plates with a sterile Pasteur pipette (8.4 mm diameter) and fulfil by tested extract or methanol. Initially, plates were incubated for 6 h at 4 °C to achieve full diffusion of the test solution in the agar medium. Then, plates were incubated at 30 or 37 °C for 24 h and zones of inhibited growth around each well were measured.

### Proximate composition

The dry matter (DM), ash, total nitrogen, fat and crude fibre content was evaluated according to the Official Methods of Analysis of AOAC International (AOAC)^31^. The moisture content of the flours was determined on the basis of weight loss during thermal drying at 105 °C until constant weight was achieved. The total ash content was determined by adding 1 g of oilseed cake flour to a crucible, incinerating it in a muffle furnace at 550 °C and determining the weight of the residue. Total nitrogen was determined by the Kjeldahl method using a nitrogen analyser. A nitrogen to protein conversion factor of 6.25 was used to calculate total protein. Fat content was determined using the Soxhlet method, in Büchi B-811 apparatus, with the use of diethyl ether after hydrolysis of the sample with 4 N HCl. Total carbohydrates were calculated by difference (100 − sum of protein, fat, ash and moisture). Crude fibre content was determined according to the Hennenberg and Stohmann method by acid hydrolysis with H_2_SO_4_ followed by alkaline hydrolysis with NaOH. Data are reported as the mean value ± standard deviation (SD) for two measurements.

### Amino acid analysis

The amino acid composition of flours was determined by ion-exchange chromatography after 23 h of hydrolysis with 6 N HCl at 110 °C. After cooling, filtering and washing, the hydrolysed sample was evaporated in a vacuum evaporator at a temperature below 50 °C. The dry residue was dissolved in a buffer of pH 2.2. The prepared sample was analysed using the ninhydrin method^32,33^. Buffers (pH 2.6, 3.0, 4.25 and 7.9) were applied. The ninhydrin solution was buffered at pH 5.5. The hydrolysed amino acids were determined using an AAA-400 analyser (INGOS, Prague, Czech Republic). A photometric detector was used, working at two wavelengths, 440 and 570 nm. A 350 × 3.7 mm column, packed with Ostion LG ANB ion exchanger (INGOS), was utilised. Column temperature was kept at 60–74 °C and the detector kept at 121 °C. Calculations were carried out relative to an external standard. No analysis of tryptophan was carried out.

### Quantitative evaluation of protein quality

The amino acid content in flours was expressed on the nitrogen basis (g per 16 g N) and it was compared to a reference protein. The amino acid pattern for high-quality protein established by the Joint Food and Agriculture Organization/World Health Organization (FAO/WHO)^34^ Committee in 1991 was chosen. Levels were calculated on the basis of the essential amino acid composition of the chemical scores (CS), according to the Mitchell and Block method^35^.

### Fatty acid analysis

Fatty acid methyl esters (FAMEs) were prepared, following the procedure described by AOAC^31^. Aliquots of 0.1 mL lipid extract for each sample were esterified with 2 mL methanolic 0.4 M NaOH solution by refluxing for 10 min at 80 °C. After addition 4 mL of BF3-etherate, the samples were boiled for 5 min. The FAMEs were extracted with hexane (2.0 mL). For drying FAMEs, 1 mL NaCl was added and set aside to make the hexane layer clear, then the upper part was poured in specific cell.

Analysis (qualitative and quantitative) of fatty acid composition was done by gas chromatography using an Agilent 7820A 9 (Santa Clara, USA) gas chromatograph. Methyl esters of fatty acids were prepared with BF_3_ in methanol as the methylating agent. An RTX-2330 capillary column (105 m, internal diameter 0.25 mm, thickness of liquid phase film 0.20 μm) was used. The detector (FID) temperature was set at 280 °C. The injection temperature was set at 260 °C. The column temperature was held at 200 °C for 21 min, then increased by 10 °C/min to 250 °C, held for 6 min and then decreased by 10 °C/min to 200 °C. Helium was used as the carrier gas. The peaks were identified based on their retention times using authentic standard fatty acids methyl esters and all samples were run in duplicate.

### Statistical analysis

All data were statistically analysed using Statistica 10.0 (StatSoft, Inc., Tulsa, OK, USA). Homogenous groups and least significant difference (LSD) values were denoted using Duncan’s multiple comparison test. The significance level was set at α = 0.05, with one-way analysis of variance (ANOVA) for three variables. To identify relationships between total polyphenols and antioxidant activity, bivariate Pearson’s correlation analysis was carried out.

## Conclusion

In the cold-press oil industry, significant amounts of by-products arise after production of the oil. Recovery of these products is important regarding economical gain. In terms of health aspects, comparative evaluation is needed to recover their potential. This study revealed that flours from oilseed by-products possess valuable potential. Important source of total phenolics with remarkable antioxidant and antimicrobial activity have evening primrose, milk thistle and corn germ flours suggesting the possibility of using them as an enrichment of existing and new food products. In analysed oilseed flours, a positive correlation was obtained between total polyphenol content and antioxidant activity (r = 0.99) as well as between total polyphenol content and antimicrobial activity of corn germ, milk thistle and evening primrose against faecalis (r = 0.66). These results could provide information about potential application of the analysed flours to substitute or complement wheat flour in new food products where these by-products could be used in the food industry to achieve economic gain.
