# Author Correction: Comparative evaluation of the antioxidant, antimicrobial and nutritive properties of gluten-free flours

**DOI:** 10.1038/s41598-021-96802-w

**Published:** 2021-08-26

**Authors:** Joanna Miedzianka, Katarzyna Drzymała, Agnieszka Nemś, Agnieszka Kita

**Affiliations:** 1 Department of Food Storage and Technology, Faculty of Biotechnology and Food Science, Wroclaw University of Environmental and Life Sciences, Chełmońskiego 37, 51‑630 Wrocław, Poland; 2 Department of Biotechnology and Food Microbiology, Faculty of Biotechnology and Food Science, Wroclaw University of Environmental and Life Sciences, Chełmońskiego 37, 51‑630 Wrocław, Poland

Correction to: *Scientific Reports* 10.1038/s41598-021-89845-6, published online 17 May 2021

The original version of this Article omitted the Funding section. The Funding section now reads:

“The publication is financed under the Leading Research Groups support project from the subsidy increased for the period 2020–2025 in the amount of 2% of the subsidy referred to Art. 387 (3) of the Law of 20 July 2018 on Higher Education and Science, obtained in 2019.”

The original Article has been corrected.

